# Lower peripheral circulation in eumenorrheic young women with premenstrual symptoms

**DOI:** 10.1186/1751-0759-1-8

**Published:** 2007-03-29

**Authors:** Tamaki Matsumoto, Takahisa Ushiroyama, Noriyuki Tatsumi

**Affiliations:** 1 Department of Health Science, International Buddhist University, 3-2-1 Gakuenmae, Habikino, Osaka, 583-8501, Japan; 2 Department of Nursing, Aino Gakuin College, Ibaragi, Osaka, Japan

## Abstract

**Background:**

A majority of women from all cultures and socioeconomic levels experience diverse psychosomatic and behavioral symptoms premenstrually, a phenomenon commonly termed premenstrual syndrome, although symptoms and discomfort levels vary from woman to woman. The underlying pathological mechanisms of premenstrual syndrome remain unknown; however, altered function or even slight disorder of the blood circulation system, which contributes to the orchestrations of the human internal environment, could cause bio-psychological changes leading to complaints and ultimately compromising a woman's overall health. The present study, therefore, investigates to what extent and how the menstrual cyclicity of peripheral circulation is associated with premenstrual symptomatology.

**Methods:**

Twenty-one eumenorrheic young women participated in this study. All subjects were investigated during the follicular and late luteal phases. Cycle phase was determined by the onset of menstruation and oral temperature and was verified by concentrations of ovarian hormones, estrone, and pregnanediol in a urine sample taken early in the morning. Peripheral circulation was evaluated with the Astrim (Sysmex, Kobe), a portable non-invasive monitoring device using the principle of near-infrared spectroscopy, which calculates the venous oxygenation index (VOI) based on the ratio of light absorption of oxyhemoglobin and deoxyhemoglobin, a proven reliable indicator of peripheral blood circulation. The Menstrual Distress Questionnaire was applied to measure physical, emotional, and behavioral symptoms accompanying the menstrual cycle of the subjects.

**Results:**

The oral temperature and urinary ovarian hormones adjusted for creatinine significantly increased in the late luteal phase in all subjects. While 10 subjects experienced no symptoms during the menstrual cycle, 11 subjects had apparent physical and psychological discomfort in the late luteal phase. We found that VOI decreased more significantly in the late luteal phase than in the follicular phase only in women with premenstrual discomfort although the symptoms were not unbearable enough to cause the disruption of daily activities.

**Conclusion:**

Several models have tried to explain the etiopathogenesis of premenstrual syndrome. Although causes and consequences remain enigmatic, our data suggest that the peripheral circulation could alter in the luteal phase, which might be partly associated with premenstrual psychosomatic symptoms in eumenorrheic young women.

## Background

Women of reproductive age have a circumlunar rhythm or a monthly cycle of the reproductive system, involving gonadotropin, ovarian hormones, and basal body temperature. During the late luteal phase, not all but a majority of women from all cultures and socioeconomic levels experience a cacophony of mind and body, that is, a regular recurrence of diverse physiological, psychological, and/or behavioral symptoms to varying degrees, which abates shortly after the onset of menstruation [1,2]. Over 150 complaints have been reported, and cardinal symptoms include: cramps, breast tenderness, swelling of extremities, irritability, depression, headaches, and food cravings [3]. This cluster of symptoms has become known as premenstrual syndrome.

**Figure 1:**
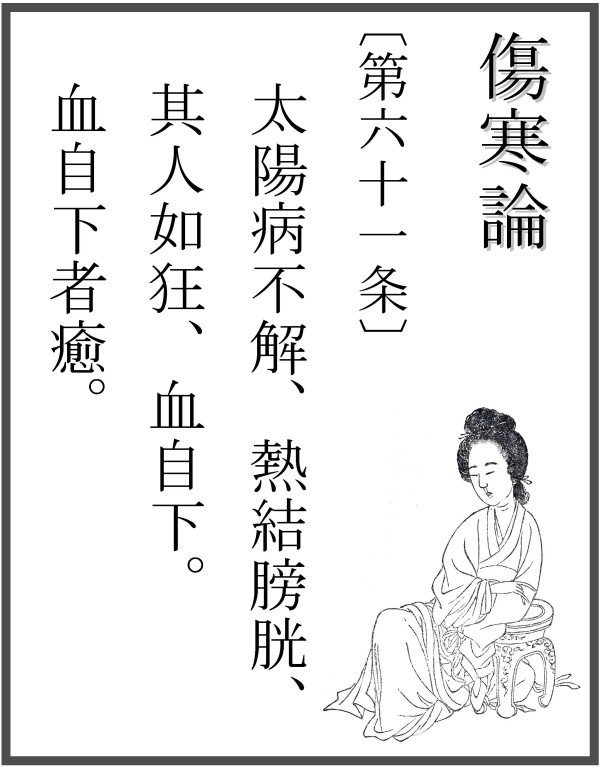
Definition of premenstrual disorders described in Chapter 61 of *Shang han lun *– the oldest Chinese practical textbook of medicine – in the early third century.

Interestingly, physicians in the East have recognized premenstrual disorders for centuries. In the early third century, the oldest Chinese practical medical textbook, *Shang han lun *(Figure 1), describes premenstrual disorders: "Women appear to have unusual conditions in the premenstrual phase while experiencing various psychosomatic symptoms including lower abdominal bloating and discomforts, which might be caused by 'oketsu' (blood stagnation) syndrome. However, they get over the symptoms after bleeding." In addition, tokaku-joki-to (tao-he-cheng-qi-tang), a type of Kampo medicine for overcoming blood stagnation or stasis, was recommended to treat premenstrual discomfort [4].

Peripheral blood circulation in capillary vessels, which account for up to 90% of total blood vessels in the body, plays a crucial role in maintaining homeostasis in the human internal environment. Thus, altered function or even slight disorder of the peripheral circulation system could induce bio-psychological changes leading to complaints and ultimately undermining overall health. Although the etiopathogenesis of premenstrual syndrome has been extensively studied [5], a paucity of information is available regarding a potential association of peripheral blood circulation and broadly ranged psychosomatic phenomena in the late luteal phase.

The Astrim (Sysmex, Kobe), a portable non-invasive monitoring device, determines hemoglobin levels by using the principle of near-infrared spectroscopy [6-8]. The device calculates the venous oxygenation index (VOI) based on the ratio of light absorption of oxyhemoglobin (HbO_2_) and deoxyhemoglobin (Hb). Previous studies have shown that the VOI serves as a potential indicator in evaluating peripheral venous oxygen metabolism and skin blood flow under different physiological circumstances [9,10]. The non-invasive portable monitoring device has several advantages, especially for clinical science research fields. It could periodically and/or constantly observe biological changes in peripheral circulation during an experiment and/or a treatment without burden or pain on subjects.

Accordingly, the present study was designed to investigate whether the VOI, an index for peripheral blood circulation, changes during the menstrual cycle in eumenorrheic young women. We further scrutinized the extent to which and the manner in which the menstrual cyclicity of peripheral circulation is associated with premenstrual symptomatology.

## Methods

### Measurement principle and function of Astrim

**Figure 2:**
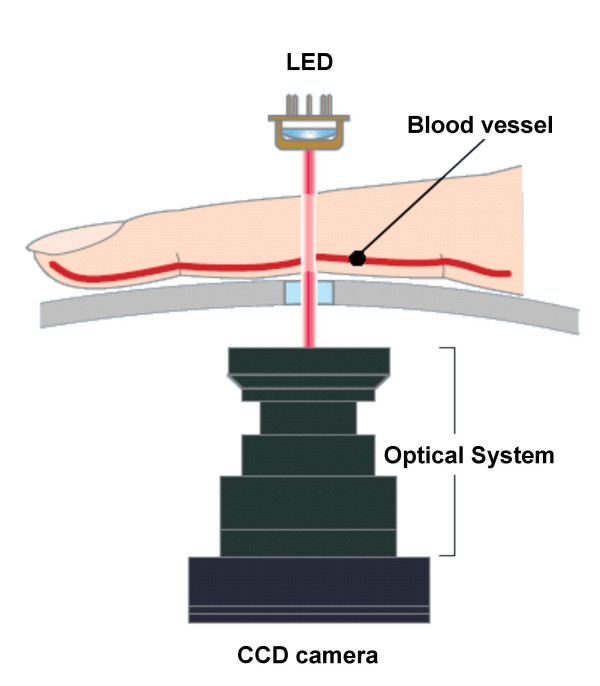
Measurement principle of the Astrim. LED (light-emitting diode), CCD (charge-coupled device).

Wavelengths in the near-infrared region are well absorbed by blood, but they penetrate tissues. The Astrim portable non-invasive monitoring device operates as follows: an infrared or near-infrared light source (880, 805, and 660 nm) is positioned over the dorsal side of a finger, and a CCD camera is positioned underneath the finger (Figure 2). The device provides an image of venous blood vessels near the skin's surface on the finger. When the finger is irradiated with light of differing wavelengths, the optical density of the vascular portions of the images at the respective wavelengths reflects the amount of light absorbed by hemoglobin. In addition, direct measurement of the vascular diameter of the optical image can evaluate the amount of blood contributing to the light absorption in the vascular portion of the image because the blood vessel has a nearly circular cross section. Thus, the blood hemoglobin content can be determined by the amount of light absorption and the estimated amount of blood.

**Figure 3:**
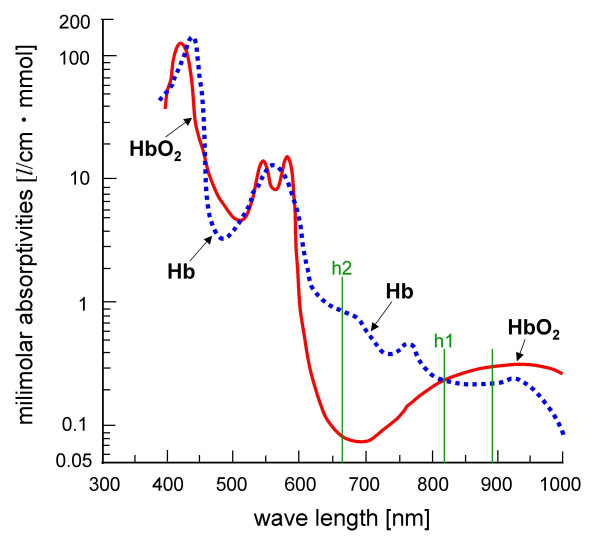
Physiological features of oxyhemoglobin (HbO_2_) and deoxyhemoglobin (Hb) represented by a thick red line and a dotted blue line, respectively, in light absorption. h1 and h2 represent the degree of light absorption of HbO_2 _and Hb at the wavelengths of 805 and 660 nm, respectively, in the near-infrared region.

The Astrim calculates venous blood vessel width and the VOI based on the ratio of light absorption of HbO_2 _and Hb. The VOI is calculated as the following equation: VOI = -h2/h1. We define h1 and h2 as the degree of light absorption of HbO_2 _and Hb at the wavelengths of 805 and 660 nm, respectively, in the near-infrared region. It should be noted that HbO_2 _and Hb absorb light equally at 805 nm, whereas at 660 nm absorption is primarily from Hb (Figure 3). The validity and reliability of the device has been demonstrated in previous studies [7,8,11,12]. The VOI has also been proven as an indicator in evaluating peripheral venous oxygen metabolism and skin blood flow under different physiological circumstances [6,9,10].

### Subjects

Twenty-one young women (mean age, 20.6 ± 0.2 years) volunteered to participate in this research. The study protocol was approved in advance by the Institutional Review Board of International Buddhist University and was performed in accordance with the Declaration of Helsinki. All subjects received an explanation of the nature and purpose of the study, and all gave their written informed consent to participate in the study. Prior to obtaining any data from experiments in the laboratory, the subjects completed a standardized health questionnaire regarding medical history, medications, current health condition, menstrual cycle (length of cycle, length of menstrual flow, and regularity of cycle), premenstrual discomfort, diet, physical activity, and lifestyle. All subjects self-reported regular menstrual cycles for at least three cycles. None of the subjects was clinically diagnosed with diabetes mellitus, hypertension, cardiovascular disease, or any other endocrine or systemic disorders. The subjects were non-obese (body mass index [BMI] < 25 kg/m^2^) and were not taking any medications, including oral contraceptives.

### Experimental Procedure

All subjects were examined on two separate occasions: once during the follicular phase, within five days after the completion of menstrual bleeding, and once during the late luteal phase, within seven days before the next menstruation. The order of testing was counterbalanced so that equal numbers of subjects were studied first in each phase. Cycle phase was determined by the onset of menstruation and oral temperature and verified by concentrations of ovarian hormones in a urine sample taken early in the morning [13].

On the days of testing, subjects came to the laboratory between 9:00 and 11:00 a.m. after having fasted overnight. All experiments were performed in the morning. The room was temperature controlled at 25°C, quiet and comfortable, with minimization of arousal stimuli. After weighing each subject, the percentage of body fat was determined by means of a bioelectrical impedance analyzer (TBF-305, TANITA, Japan). BMI was calculated as body weight divided by height squared.

The subjects rested for 10 minutes at room temperature while seated in a comfortable chair. The left middle finger was then placed on the detection probe of the device, and hemoglobin levels, venous blood vessel width, and VOI were automatically determined. The surface-skin temperature of the finger was also measured by Digital Thermometer (Tx10, YOKOKAWA). Each subject underwent five trials at 30-second intervals. During the experiment, the finger remained in the same position on the detection probe.

After the subjects completed the experiment for peripheral blood circulation, each filled out the Menstrual Distress Questionnaire (MDQ) [14] which evaluated physical, emotional, and behavioral symptoms accompanying the menstrual cycle. The MDQ consists of 46 symptoms in eight categories: pain, concentration, behavioral change, autonomic reactions, water retention, negative affect, arousal, and control. The MDQ has been widely used in menstrual cycle research [13,15], and a recent study has reconfirmed the validity and applicability of the questionnaire for evaluating menstrual symptoms [16].

### Urinary analysis

Each subject collected urine at the first urine void in the morning. Refrigerated 10-mL aliquots of urine were immediately frozen and stored at -20°C until assay. Urine samples were then analyzed for estrone conjugate (E1C) and pregnanediol-3-glucuronide (PdG) by radioimmunoassay as described by Munro et al. [17] and De Souza et al. [18]. E1C and PdG were both indexed to creatinine (Cr) excretion in the same sample to control for variations in urine volume. E1C and PdG are expressed as nanograms and micrograms per mg Cr, respectively.

### Statistical analyses

All data are expressed as mean ± SE. Paired *t*-test was performed to assess statistical differences of variables between the follicular and the late luteal phases. A comparison between two groups was made with Student's unpaired *t*-test. *P *values < 0.05 were considered statistically significant. All statistical analysis was performed using a commercial software package (SPSS version 13.0 for Windows, SPSS inc., Illinois).

## Results

### Clinical characteristics of subjects

The length of the menstrual cycle and the duration of menstrual flow of all subjects during the study were 27.9 ± 0.7 days and 6.1 ± 0.2 days, respectively. The days of the experiments were 10.6 ± 0.3th day in the follicular phase and 25.3 ± 0.7th day in the late luteal phase from the first day of menstruation.

Concerning menstrual cycle symptomatology, some subjects had apparent physical and psychological discomfort in the late luteal phase while others experienced few symptoms. To scrutinize the potential influence of premenstrual discomfort on the peripheral circulation, we divided the subjects into two groups based on the increase in scores on the MDQ while referring to the findings from our recent study [13], i.e., 11 subjects in the Control Group (less than one point) and 10 subjects in the premenstrual symptoms (PMS) Group (greater than 14 points). It should be mentioned, that according to the results of a standardized health questionnaire and individual interviews, none of the subjects in the PMS Group experienced distressing physical, psychological, and/or behavioral changes of sufficient severity to result in deterioration of interpersonal relationships and/or interference with normal activities. In addition, no subjects in the present study sought medical treatment.

Table 1 shows clinical characteristics of the Control and PMS Groups. Basal body temperature (*p *< 0.05) and concentration of E1C (*p *< 0.05) and PdG (*p *< 0.01) in urine were more elevated in the late luteal phase than in the follicular phase in both groups. Body weight (*p *< 0.01) and BMI (*p *< 0.01) significantly increased in the late luteal phase compared to the follicular phase only in the PMS Group. Group comparison revealed no significant difference in ovarian hormones or in body composition during the menstrual cycle between the Control and PMS Groups.

**Table 1:** Clinical characteristics of subjects in the follicular and late luteal phase

	Control Group (n = 10)		PMS Group (n = 11)	
	
	Follicular	Luteal	Follicular	Luteal
Basal body temperature (°C)	36.04 ± 0.10	36.51 ± 0.14*	36.36 ± 0.08	36.43 ± 0.11*
Estrone conjugates (ng/ml Cr)	11.1 ± 2.3	20.4 ± 4.2*	12.6 ± 1.5	19.6 ± 6.2*
Pregnanediol-3-glucuronide (μg/ml Cr)	0.6 ± 0.2	3.7 ± 0.6**	0.4 ± 0.04	2.1 ± 0.4**
Weight (kg)	52.3 ± 1.7	52.5 ± 1.8	53.9 ± 1.8	54.6 ± 1.8**
Body Mass Index (kg/m^2^)	20.4 ± 0.4	20.4 ± 0.5	20.2 ± 0.6	20.5 ± 0.6**
Body fat (%)	25.9 ± 1.0	25.8 ± 1.0	24.8 ± 1.1	25.2 ± 1.1

Cr: creatinine

Values are means ± SE. **p < 0.01; *p < 0.05 (Follicular phase vs. Late luteal phase)

### Bio-psycho-behavioral symptoms

**Figure 4:**
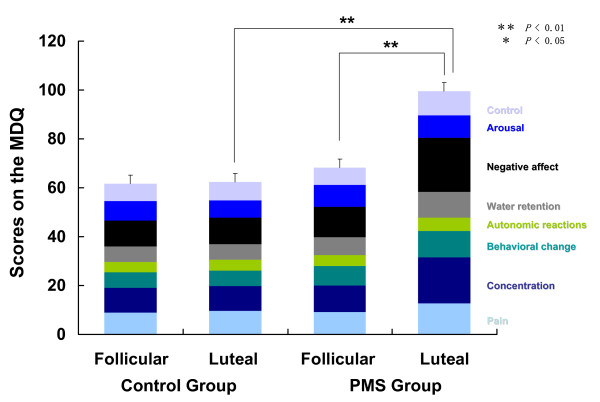
Comparison of scores on the menstrual distress questionnaire (MDQ) between the follicular and late luteal phases in the Control and PMS Groups. Results are expressed as mean ± SE. for each group. ** *p *< 0.01; * *p *< 0.05.

Figure 4 represents MDQ scores in the follicular and the late luteal phases in the Control and PMS Groups. No significant increase was detected either in subscores or in total scores between the menstrual phases in the Control Group. In the PMS Group, however, total scores markedly increased from the follicular to the late luteal phase (*p *< 0.01). Scores of all factors were greater in the late luteal phase than those in the follicular phase and significant changes were detected in the following factors: pain (*p *< 0.05), concentration (*p *< 0.01), behavioral change (*p *< 0.05), water retention (*p *< 0.05), negative affect (*p *< 0.01), and control (*p *< 0.01).

Group comparison revealed no significant differences in subscores or in total scores on the MDQ between the Control and PMS Groups in the follicular phase. The total scores on the MDQ in the late luteal phase were, however, significantly greater in the PMS Group than in the Control Group. All subscores of the late luteal phase were higher in the PMS Group compared to those in the Control group, and statistical analysis further demonstrated significant differences in pain (*p *< 0.05), concentration (*p *< 0.01), behavioral change (*p *< 0.01), water retention (*p *< 0.01), negative affect (*p *< 0.01), and control (*p *< 0.01) factors.

### Peripheral circulation

According to our previous study investigating the validity and reliability of the Astrim [9], data of each parameter obtained from five trials were averaged for statistical analysis. No significant difference in hemoglobin levels was found between the follicular and the late luteal phase in the Control Group (11.8 ± 0.5 vs. 11.9 ± 0.4 g/dl). In the PMS Group, hemoglobin levels were lower in the late luteal phase compared to the follicular phase (12.1 ± 0.5 vs. 11.6 ± 0.9 g/dl) without statistically significant differences. The surface-skin temperature did not differ between the menstrual phases in the Control Group (31.8 ± 1.1 vs. 31.2 ± 0.4°C). No significant effect of the menstrual cycle was found in the PMS Group (32.3 ± 0.6 vs. 31.2 ± 1.0°C) although the temperature was lower in the late luteal phase.

**Figure 5:**
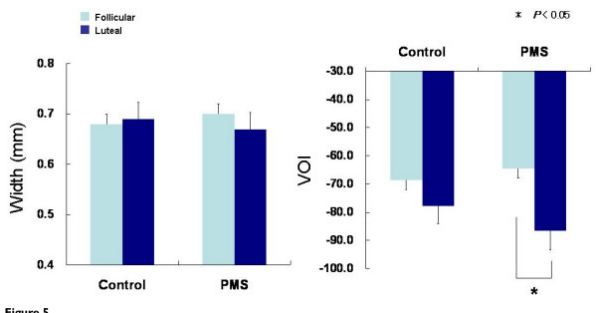
Comparison of the venous blood vessel width (Width) and the venous oxygenation index (VOI) between the follicular and late luteal phases in the Control and PMS Groups. Results are expressed as mean ± SE. for each group. * *p *< 0.05.

As to the parameters of peripheral blood circulation detected by the Astrim (Figure 5), no significant change was found in the venous blood vessel width between the menstrual phases in the Control Group. The width was smaller in the late luteal phase compared to the follicular phase in the PMS Group, but the difference did not reach statistical significance. VOI decreased in the late luteal phase in both groups. The degree of change in VOI from the follicular to the late luteal phase was more apparent in the PMS Group and the statistical analysis verified the significant difference between the two phases (*p *< 0.05).

## Discussion

Premenstrual syndrome has been recognized worldwide. According to epidemiological reports, including ones in Japan, approximately 70 to 90 percent of women of childbearing age experience at least some uncomfortable premenstrual symptomatology, with from 2 to 10 percent having disabling, incapacitating symptoms [2,19]. Despite its high prevalence, no specific symptoms or signs appear, nor are any recognizable anatomical factors identified in women suffering from premenstrual syndrome. This leads to difficulty in appropriately diagnosing the pathophysiological conditions and/or of offering effective medical treatments.

From the perspective of traditional Chinese medicine, now called Kampo Medicine, oketsu (blood stagnation) and suidoku (disorders of water metabolism) have been considered main factors in premenstrual syndrome. In addition, premenstrual dysphoric disorder (PMDD), the most severe type of premenstrual symptomatology, could be attributable to inadequate blood flow induced by oketsu, which results in qi stagnation including diverse psychosomatic symptoms, i.e., pain, oppression in the chest, abdominal distention, melancholia, depressive mood, heavy feeling of the head, difficulty in breathing, sensation of swelling, and stiffness in the extremities [4,20,21]. Clinical studies have shown that tokaku-joki-to (tao-he-cheng-qi-tang), a type of Kampo medicine for overcoming blood stagnation, was a first-line therapy to treat premenstrual discomfort, and kami-shoyo-san (jia-wei-xiao-yao-san), keishi-buku-ryogan (gui-zhi-fu-ling-wan), and toki-shakuyaku-san (dang-gui-shao-yao-san), the typical herbal preparations improving blood circulation, were also effective in alleviating and ameliorating unidentified symptoms in the premenstrual phase [4,20]. These findings suggest that a deteriorated blood circulatory system could be associated with premenstrual symptomatology; however, little substantial evidence has been reported thus far.

In the present study, we used the VOI, a parameter detected from the non-invasive monitoring devise with optical technology, to investigate a potential etiologic association between the peripheral circulation and premenstrual symptomatology. As to the physiological significance and features of VOI, a study at the Central Research Laboratory of the Sysmex Corporation reported a highly significant positive correlation between venous oxygen saturation and the VOI [9]. Ozawa et al. [10] have shown that the VOI as well as blood flow evaluated by the laser Doppler blood-flowmeter dramatically decreased against cold exposure. In addition, recent research [6,9] has reported that the VOI markedly decreased during the brachial 150 mmHg occlusion shortly after the handgrip exercise. After releasing the occlusion, however, VOI increased and returned to its level prior to the handgrip exercise due to the restoration of blood circulation, which contributed to improving oxygen supply. These findings indicated that the VOI serves as an effective indicator of peripheral venous oxygen metabolism and skin blood flow under different physiological circumstances.

By applying the parameter to the present research, we have found VOI decreased more significantly in the late luteal phase than in the follicular phase only in the PMS Group. In addition, the women of this group tend to possess lower VOI in the late luteal phase than the Control Group. It should be restated that scores of premenstrual symptoms were markedly higher in the PMS Group than in the Control Group, but the symptoms were not as severe as to cause disruption of daily activities or to need medical consultations, according to the questionnaires and individual interviews. It would be of interest to scrutinize whether the VOI would markedly worsen in women suffering from distressing and unbearable premenstrual symptoms including PMDD when compared to healthy women without symptoms.

Fluctuations in the ovarian hormones along the menstrual cycle have been suggested to influence vasoconstriction in peripheral blood vessels [22]. The present investigation, however, detected no significant differences in ovarian hormone concentration in either the follicular or late luteal phase between the two groups. Simpson [23] formally hypothesized that capillaries could be smaller in size in women with premenstrual syndrome than asymptomatic controls due to the altered noradrenergic and/or thyroid functions. Girdler et al. [24] have revealed that women with PMDD had significantly elevated norepinephrine and total peripheral resistance, which could affect peripheral blood circulation, at rest and during mental stressors compared with control subjects. Future research will be needed to explicate exact neurophysiological and/or hormonal effects of the menstrual cycle. The present study, however, indicates that peripheral blood circulation decreased in the symptomatic late luteal phase from the non-symptomatic follicular phase even in eumenorrheic young women who were not diagnosed as having serious premenstrual disorders, but experienced a substantial increase in diverse psychosomatic symptoms premenstrually.

## Conclusion

Premenstrual disorders can affect a woman at any stage in her reproductive life – beginning around age 14, or about 2 years after menarche, and persist until around age 51, when menopause typically occurs [25]. In this stage of life, a woman's living environment as well as physiological conditions can change dramatically. Several theories have tried to explain the etiopathogenesis of premenstrual syndrome [2,4,5], but the underlying biomechanisms with the complex web of bio-psycho-socio-ethical factors remain enigmatic. Although causes and consequences continue to elude, the present study provides additional intriguing evidence that the occurrence of premenstrual symptomatology is associated with an altered functioning of peripheral circulatory system in the late luteal phase. This study has also shown the applicability of the non-invasive monitoring devise using the principle of near-infrared spectroscopy. Since it is non-invasive and imposes no burden or pain during an examination, the device could be utilized in diverse psycho-physiological research and clinical situations, such as monitoring patients' conditions and promoting women's health.

## Competing interests

The author(s) declare that they have no competing interest.

## Authors' contributions

TM conceptualised and designed the study, collected and analysed the data, performed the statistical analysis, interpreted the results, and drafted the manuscript. TU provided substantial clinical evidence of Kampo medicine from his gynaecological research and constructive suggestions to develop the present study, and helped to draft and revise the manuscript. NT contributed by inventing the Astrim, a non-invasive monitoring device, and participated in the design and coordination of the present study. All authors read and approved the final manuscript.
